# Patch-Based Super-Resolution of MR Spectroscopic Images: Application to Multiple Sclerosis

**DOI:** 10.3389/fnins.2017.00013

**Published:** 2017-01-31

**Authors:** Saurabh Jain, Diana M. Sima, Faezeh Sanaei Nezhad, Gilbert Hangel, Wolfgang Bogner, Stephen Williams, Sabine Van Huffel, Frederik Maes, Dirk Smeets

**Affiliations:** ^1^icometrix, R&DLeuven, Belgium; ^2^Department of Electrical Engineering-ESAT, STADIUS Center for Dynamical Systems, Signal Processing and Data Analytics, KU LeuvenLeuven, Belgium; ^3^Centre for Imaging Sciences, University of ManchesterManchester, UK; ^4^High Field MR Centre, Department of Biomedical Imaging and Image-guided Therapy, Medical University of ViennaVienna, Austria; ^5^Christian Doppler Laboratory for Clinical Molecular MR ImagingVienna, Austria; ^6^ImecLeuven, Belgium; ^7^Department of Electrical Engineering-ESAT, PSI Medical Image Computing, KU LeuvenLeuven, Belgium; ^8^BioImaging Lab, Universiteit AntwerpenAntwerp, Belgium

**Keywords:** super-resolution, up-sampling, magnetic resonance spectroscopy imaging, patch-based, multiple sclerosis

## Abstract

**Purpose:** Magnetic resonance spectroscopic imaging (MRSI) provides complementary information to conventional magnetic resonance imaging. Acquiring high resolution MRSI is time consuming and requires complex reconstruction techniques.

**Methods:** In this paper, a patch-based super-resolution method is presented to increase the spatial resolution of metabolite maps computed from MRSI. The proposed method uses high resolution anatomical MR images (T1-weighted and Fluid-attenuated inversion recovery) to regularize the super-resolution process. The accuracy of the method is validated against conventional interpolation techniques using a phantom, as well as simulated and *in vivo* acquired human brain images of multiple sclerosis subjects.

**Results:** The method preserves tissue contrast and structural information, and matches well with the trend of acquired high resolution MRSI.

**Conclusions:** These results suggest that the method has potential for clinically relevant neuroimaging applications.

## 1. Introduction

Magnetic resonance spectroscopy imaging (MRSI) of the brain provides information on the chemical composition of tissues and may reveal underlying metabolic changes that are not visible on conventional magnetic resonance imaging (MRI) such as T1-weighted MRI. For example in multiple sclerosis (MS), MRSI can show the extent of damage in normal appearing white matter and therefore is more sensitive in detecting pathological changes compared to conventional MRI (Filippi and Agosta, 2010; Rovira et al., 2013). Additionally, metabolite concentrations in normal appearing brain tissue are better correlated with clinical scores than MRI lesion load (Sajja et al., 2009).

Despite the fact that MRSI provides attractive complementary information with respect to conventional MRI, it is still not widely used in practice. Possible reasons include (a) low signal to noise ratio, (b) poor spatial resolution, (c) prolonged acquisition time, (d) lack of standardized protocols, (e) and limited quality control. Since the metabolite concentrations are very low, a large voxel size is required in order to capture sufficient signal, resulting in a poor spatial resolution (typically around 1 cm^3^). Additionally, this results in a broad point spread function which further causes spectral signal leakage in the neighboring voxels. This limits the ability to differentiate among spatial features of interest, e.g.,between different tissue types. High spatial resolution MRSI cannot be acquired in the clinical routine with standard acquisition sequences as it requires a long scanning time, e.g.,in the range of tens of minutes.

Therefore, there is a need to design alternative, non-conventional approaches to create high resolution MRSI-based metabolic images within a limited acquisition time. The acquisition time can be reduced by optimising the conventional phase encoded MRSI protocol (Posse et al., 1995; Pohmann et al., 1997; Adalsteinsson et al., 1998) or by using parallel imaging reconstruction based techniques (Dydak et al., 2001; Lin et al., 2007; Otazo et al., 2007; Banerjee et al., 2009) or combination of both Dreher et al. (2011), and Posse et al. (2013) for a detailed review. Conventional MRSI protocols contain a series of periodic spatial and spectral encoding schemes. These could be optimized by reading out the spatial information during spectral acquisition. This optimized technique forms the basis of the echo planar spectroscopic imaging (EPSI) (Posse et al., 1995) which provides an improved spectral and spatial resolution compared to conventional MRSI protocols for fields up to 3T. Derived from the EPSI technique, spiral MRSI (Adalsteinsson et al., 1998; Andronesi et al., 2012; Bogner et al., 2014) uses a spiral trajectory in k-space, which allows faster acquisition than EPSI however at the cost of increased complexity in the data reconstruction. Parallel imaging reconstruction techniques are based on the principle of acquiring multiple undersampled k-space data using phased array coils. This undersampled data is then reconstructed into a single image using techniques like SENSE (Pruessmann et al., 1999) or GRAPPA (Griswold et al., 2002). Since optimized MRSI protocols like EPSI and parallel imaging are independent in nature, they can be combined to further reduce the acquisition time (Dreher et al., 2011). This reduction in the acquisition time generally comes at the cost of decreased signal to noise ratio (SNR) in the data. In spite of advances in speeding up the MRSI acquisition these techniques are yet not clinically feasible as the time required to attain the spatial resolution of conventional MRI sequences such as T1-weighted imaging still remains very high, although approaches that combine phase-encoding and EPSI (Ma et al., 2017) appear quite promising.

An ultra-high magnetic field strength such as 7T (used for non-routine research) offers more SNR, but the inherent limitations (e.g., related to specific absorption (SAR) or *B*_0_/*B*_1_ homogeneity) have complicated high-resolution MRSI acquisition until the recent years. Free induction decay (FID) MRSI (Bogner et al., 2012; Považan et al., 2015; Strasser et al., 2013) with short acquisition delays was proposed to avoid SAR-intensive localization schemes and SNR-loss due to *T*2^*^ decay. The application of parallel imaging like SENSE (Zhu et al., 2013; Kirchner et al., 2015), GRAPPA (Hangel et al., 2015), or CAIPIRINHA (Strasser et al., 2016) allowed acceleration factors up to 9. Combining parallel imaging with a short TR of 200 ms, the acquisition of high-resolution MRSI within a 128 × 128 *mm*^2^ field of view with a voxel volume of 23 μ*L* and full slice coverage in around 10 min was successfully demonstrated in Hangel et al. (2016).

An alternative is to increase the MRSI resolution by super-resolution (SR) techniques. Super resolution methods include k-space based reconstruction methods that improve the spectral quality of reconstructed high resolution MRSI data using high resolution spatial features, such as edges, from other imaging modalities, in particular T1-weighted MRI (for a detailed review see Kasten et al., 2016). Super-resolution methods can be categorized into two sub-groups: methods based on a linear regression framework, or on a Bayesian framework. Linear regression based SR methods (LRSR) assume that the acquired data can be explained by a linear combination of a set of independent variables. Therefore, they aim at optimising the coefficients of these independent variables such that the error between the predicted and the actual measurements is minimized. One such method is spectral localization by imaging (SLIM) (Hu et al., 1988) which assumes that the high resolution MRSI consists of a linear combination of *L* anatomical compartments that are spectrally homogeneous. Although easy to implement, the assumption of spectral homogeneity may not hold true. To cope with this, two extensions have been proposed: generalized SLIM (GSLIM) (Liang and Lauterbur, 1991) and SLIM with explicit B_0_ field inhomogeneity compensation (BSLIM) (Khalidov et al., 2007). GSLIM uses spatial Fourier harmonics to absorb any spatially dependent spectral variations and BSLIM assumes that the spectral variations are solely due to local (static) field inhomogeneity, therefore, requires an additional B_0_ map for its correction. It has been observed that simple LRSR methods not always result in physically plausible solutions, therefore, an additional term commonly known as “regularizer” is added to the optimization problem so that the reconstruction remains well-behaved. For example, in Jacob et al. (2007), a local B-spline basis function is added as a regularizer to the GSLIM model to allow local intensity variations within each compartment. In Haldar et al. (2008), a smoothness regularization term (controlled by pre-computed anatomically derived weight factors) has been added to penalize the local spectral variation between neighboring voxels. The second sub-group of methods are based on Bayesian theory and model the reconstruction in the k-space as a likelihood function where the anatomical information acts as prior knowledge to estimate the optimized model parameters via the expectation-maximization algorithm. For example in Bao and Maudsley (2007), the likelihood function consists of a combined spectral-spatial model where the tissue segmentations acts as prior information in estimating additional high frequencies in k-space. As an extension of this work, the likelihood model in Kornak et al. (2010) also addresses the spectral fitting problems and additionally uses prior information on the relationship between tissue segmentation and spatial metabolite distribution. These Bayesian based models are very complex in nature and have many variables to be optimized that often result in locally optimal solutions.

Contrary to the k-space based super-resolution approaches presented above, patch-based super-resolution (PBSR) methods have been used to reconstruct high resolution T2 and diffusion weighted MR images (Manjón et al., 2010a,b; Rousseau and The Alzheimer's Disease Neuroimaging Initiative, 2010; Coupé et al., 2013) in the spatial domain using a high resolution T1-weighted image as reference. PBSR methods are based on the principle of image redundancy (ability to find similar patches in an image) and aim at finding similar voxels [defined using self similarity or using the high resolution image (e.g., T1-weighted image)] in the neighborhood of the central voxel that could be used to guide the reconstruction of the central voxel. As opposed to classical interpolation methods (nearest neighbor, linear interpolation and B-splines), PBSR method provides better tissue contrast and thus yields better image quality reconstructions.

In view of good performance of PBSR methods in upsampling T2 and diffusion weighted MR images, we propose a multi parametric patch-based super-resolution method that acts directly on quantified MRSI metabolite maps from low-resolution MRSI data. In addition to the conventionally used high resolution T1-weighted image, a FLAIR image is also used for lesion segmentation in multiple sclerosis subjects. The reconstruction process is guided by the intensities of T1-weighted and FLAIR images along with the brain segmentation, thus providing better estimation of brain tissue boundaries. To the best of our knowledge, a PBSR technique has never been applied before for upsampling MRSI-based metabolic images. We compare our method against the classical upsampling methods (nearest neighbor, linear interpolation and B-splines) on (a) an acetate image from a phantom, which contains different acetate proportions in different spatial locations, (b) simulated MS brain and quantified N-acetylaspartate images and (c) real images of MS patients with quantified N-acetylaspartate and myo-inositol images.

## 2. Methods

**Figure 1:**
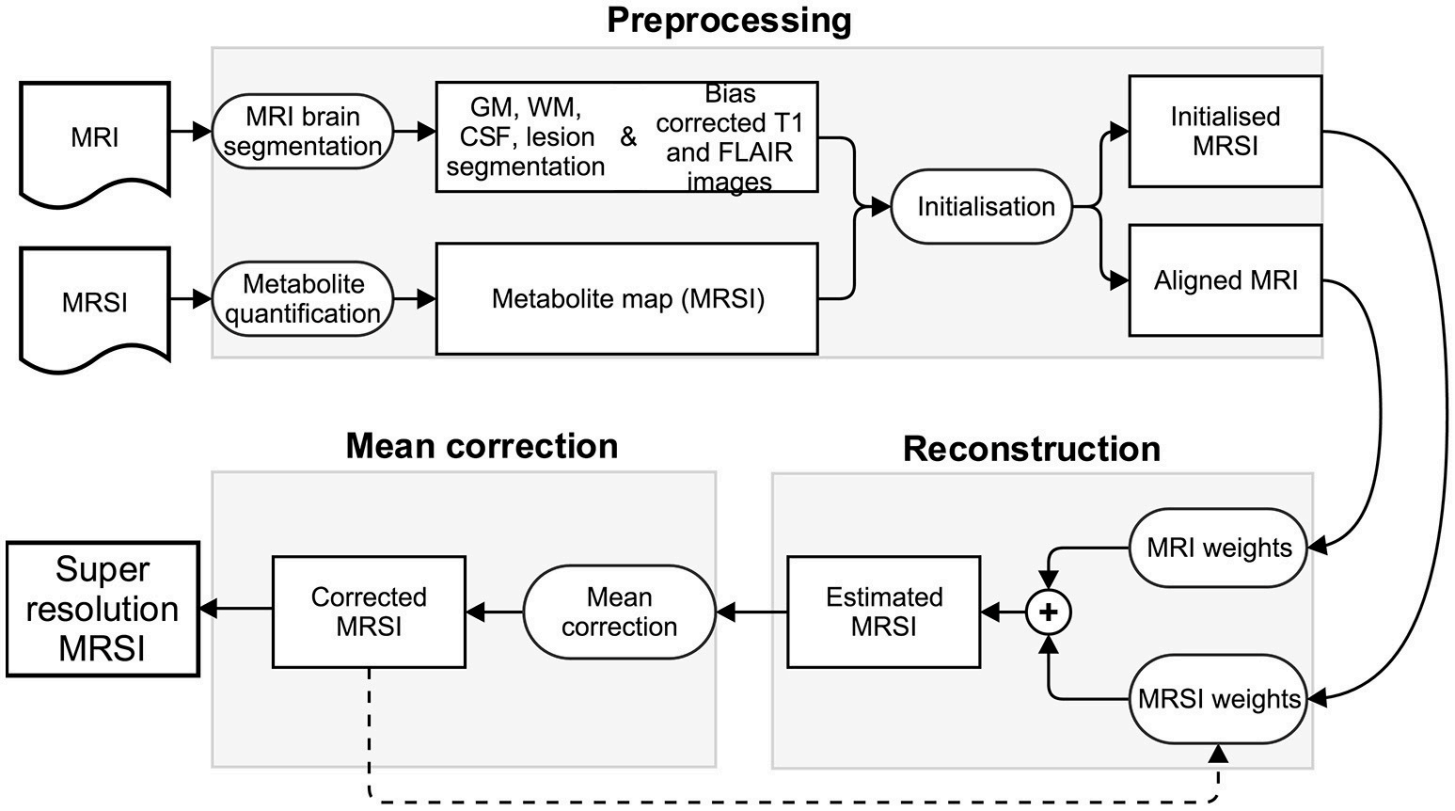
**Schematic representation of patch-based super-resolution pipeline**.

The patch-based super-resolution pipeline upsamples the low resolution quantified MRSI metabolite map using the high resolution T1-weighted and FLAIR MR images. In the next sections, we describe the pipeline that has three steps: (1) a preprocessing step, including (a) MRI brain tissue segmentation, (b) metabolite quantification, (c) super-resolution initialization, (2) a reconstruction step that estimates the metabolite concentration at each higher resolution MRSI voxel using the tissue segmentations along with image intensities of bias corrected T1-weighted and FLAIR images, (3) a mean correction step that rectifies the estimated metabolite concentration in every high resolution voxel by taking into account a point spread function (PSF) and the error toward the corresponding lower level voxel's metabolite value. The method iterates between step 2 and step 3 such that the corrected metabolite concentration map from previous iteration is used to initialize the metabolite concentration prior map for the current iteration. The convergence of our method is detected when the relative metabolite concentration difference between the current and previous iteration is negligible. It takes generally five iterations for the algorithm to converge. An overview of the pipeline is shown in Figure 1.

### 2.1. Preprocessing

#### 2.1.1. MRI brain tissue segmentation

The anatomical brain MR images are segmented into gray matter (GM), white matter (WM), cerebrospinal fluid (CSF) and lesions using **MSmetrix** (Jain et al., 2015). The method iteratively segments the T1-weighted image into GM, WM, and CSF, segments the WM lesions on the FLAIR image as an outlier to normal brain using Mahalanobis distance, and performs lesion filling in the T1-weighted image to improve tissue segmentation at next iteration. After convergence, soft segmentations of GM, WM, and CSF are created together with binary lesion segmentation. In addition, bias corrected T1-weighted and FLAIR images are also produced.

#### 2.1.2. Metabolite quantification

The acquired MRSI data, after Fourier transformation from k-space to the spatial domain, can be fitted in a voxel-by-voxel fashion using a simulated metabolite basis set, matching the acquisition parameters (e.g., echo time) of the given signals. The fitting can be based either on time-domain MRSI signals, or after Fourier transformation to the frequency domain. In this work we used the SPID software^1^ (Van Cauter et al., 2013) and LCModel^2^ for metabolite quantification. The output consists of a set of metabolite images (“metabolite maps”), as many as metabolites in the basis set, and having the same spatial resolution as the input MRSI grid.

#### 2.1.3. Super-resolution initialization

The super-resolution pipeline is initialized by (1) upsampling the low resolution metabolite map using linear interpolation (scale factor of 2). This forms the starting value of the high resolution MRSI metabolite map, (2) by aligning the MRI and MRSI images. As the MRI and MRSI images are acquired on the same scanner and in a sequential manner, their affine transformations are used to project the MRI image along with the tissue segmentations into the space of the upsampled MRSI image. The total affine transformation that relates these two image spaces is defined as:

(1) $$\begin{array}{r} Total_{affine}=MRSI_{affine}^{-1}.MRI_{affine} \end{array}$$

### 2.2. Patch-based super-resolution

#### 2.2.1. Background

Let *y* be a noise-free low resolution metabolite map and *x* be the unknown corresponding high resolution metabolite image defined over a high resolution voxel space Ω. Theoretically,

(2) $$\begin{array}{r} y=H\left(x\right) \end{array}$$

where *H* is a known blur and downsampling operator. In order to reconstruct *x* given *y*, the reconstruction model is typically formulated as an Euler Lagrange energy minimization problem:

(3) $$\begin{array}{r} \hat{x}=arg\;min\left\{\underset{\text{least squares fitting}}{\underset{︸}{||y-H\left(x\right)|{|}^{2}}}+\;\lambda \underset{\text{regularizer}}{\underset{︸}{\text{R}(\text{x})\;}}\right\} \end{array}$$

where the parameter λ controls the amount of regularization and the *R(x)* term preserves geometry and regularity and is defined as:

(4) $$\begin{array}{r} \text{R}(\text{x})\;=\underset{i\in \Omega }{\sum }||x_{i}-E\left(x_{i}|\zeta_{i}\right)|{|}^{2} \end{array}$$

*E*(*x*_*i*_|ζ_*i*_) is the conditional reconstruction of *x*_*i*_ based on voxel intensity values in the “search volume” ζ_*i*_.

We assume that the term *E*(*x*_*i*_|ζ_*i*_) is consistent under strictly stationary conditions, i.e., this stationary condition amounts to saying that, as the resolution of the image increases, there are many similar patches for all the details of the image. Also, when the regions move far from each other, their correlation decreases (Buades et al., 2005). Both assumptions are approximately met in the case of MRSI. If *E*(*x*_*i*_|ζ_*i*_) is modeled under these assumptions, then *x*_*i*_ → *E*(*x*_*i*_|ζ_*i*_) which means *R(x)* → 0 (Roussas, 1990). Therefore, the two terms of the energy functional can be decoupled and asymptotically approximated as the reconstruction and mean correction steps of the iterative super-resolution algorithm.

**Algorithm:** SR(*y* = low res image; *x* = starting value for high res image)

$\begin{array}{l} \text{while}\;|x_{i}^{t}-x_{i}^{t-1}|\;>\;\epsilon ,\;\forall \;x_{i}\in \;x\\ \{\begin{array}{l} Reconstruction:x_{i}^{t+1}=E(x_{i}|\zeta_{i}),\;\forall \;x_{i}\in \;x\\ Mean\;correction:{\hat{x}}_{i}^{t+1}=x_{i}^{t+1}-\underset{\text{error}}{\underset{︸}{\left(y_{p}-H(x_{i}^{t+1})\right)}} \end{array} \end{array}$

where *p* is the index of the corresponding voxel at low resolution of which *x*_*i*_ is a part.

#### 2.2.2. Reconstruction

The reconstruction term, *E*(*x*_*i*_|ζ_*i*_) in its general form is defined as:

(5) $$\begin{array}{r} E\left(x_{i}|\zeta_{i}\right)=\underset{j\in \zeta_{i}}{\sum }w\left(x_{i},x_{j}\right)x_{j} \end{array}$$

where the weight *w*(*x*_*i*_, *x*_*j*_) defines the contribution of neighborhood voxel *x*_*j*_ in the reconstruction of *x*_*i*_. Mathematically, the general form of *w*(*x*_*i*_, *x*_*j*_) is defined as Coupé et al. (2013):

(6) $$\begin{array}{r} w\left(x_{i},x_{j}\right)=\frac{1}{Z_{i}}e^{-\frac{1}{2N}{\left(\frac{\left||\hat{N_{i}}-\hat{N_{j}}|\right|}{h_{i}}\right)}^{2}} \end{array}$$

where *Z*_*i*_ is a normalization constant such that $\sum_{j\in \zeta_{i}}w(x_{i},x_{j})=1$, $\hat{N_{i}}$ is the intensity vector of the local neighborhood *N*_*i*_ of length *N*, *h*_*i*_ is the standard deviation of neighborhood voxel intensities in *N*_*i*_. *N*_*i*_ is empirically defined as a cube of size 8 × 8 × 8 voxels around the centre voxel *x*_*i*_. From Equation (6), we observe that if $\hat{N_{j}}$ is similar to $\hat{N_{i}}$, more weight is given to *x*_*j*_ in the reconstruction of *x*_*i*_. Since we use both high resolution MRI (T1-weighted and FLAIR) and low resolution MRSI, the weights are refined for both modalities taking into account the prior information:

##### Refining the weights for high resolution MR images

*w*_*MRI*_(*x*_*i*_, *x*_*j*_) are defined such that the weights in Equation (6) accommodate the prior knowledge on brain tissue segmentations that were down-sampled to match the MRSI resolution:

(7) $$\begin{array}{l} w_{MRI}\left(x_{i},x_{j}\right)=\frac{1}{Z_{i}.K}\overset{K}{\underset{k}{\sum }}p_{i,k}.p_{j,k}.e^{-\frac{1}{2N}{\left(\frac{\left||{\hat{N}}_{i}-{\hat{N}}_{j}|\right|}{h_{i}}\right)}^{2}} \end{array}$$

where *k* ∈ *K* = {GM, WM, CSF}, *p*_., *k*_ denotes the probability that the voxel belongs to a particular tissue class *k*.

##### Refining the weights for low resolution MRSI

The *w*_*MRSI*_(*x*_*i*_, *x*_*j*_) weights have the form (6), and the only unknown term is *h*_*i*_. Assuming white noise Gaussian distribution, *h*_*i*_ can be calculated using the pseudo residual ϵ_*i*_:

(8) $$\begin{array}{r} \epsilon_{i}=\sqrt{\frac{6}{7}}\left(x_{i}-\frac{1}{6}\underset{n\in N_{i}^{*}}{\sum }x_{n}\right) \end{array}$$

where $N_{i}^{*}$ is the 6-neighborhood (4 in-plane and 2 out-of-plane neighbors) of *x*_*i*_. Now,

(9) $$\begin{array}{r} h_{i}^{2}=\frac{1}{M}\overset{M}{\underset{i=0}{\sum }}\epsilon_{i}^{2} \end{array}$$

where *M* is the number of voxels in ζ_*i*_ that have non-zero weight in the reconstruction of *x*_*i*_.

After computing weights for both modalities, *E*(*x*_*i*_|ζ_*i*_) is defined similar to Rousseau and The Alzheimer's Disease Neuroimaging Initiative (2010):

(10) $$\begin{array}{l} E(x_{i}|\zeta_{i})=\sum_{j\in \zeta_{i}}\{(1-\alpha (x_{i}))w_{MRI}+\alpha (x_{i})w_{MRSI}\}x_{j} \end{array}$$

where α(*x*_*i*_) is a weighing term between the MRI and MRSI weights. The choice of α(*x*_*i*_) is application specific. For example, in case of non-pathological studies, the reconstruction process could be driven by MRI only, and therefore, α(*x*_*i*_) can be set to zero. In case of pathological cases like MS, MRSI is more sensitive to pathological changes such as lesions compared to MRI. As we focus on MS subjects in this paper, α(*x*_*i*_) is defined using binary lesion segmentation which was obtained in the preprocessing step (see Section 2.1.1). In particular, the weight α is defined for each voxel *x*_*i*_ such that the α(*x*_*i*_) = 1 implies that it is a lesion and thus the reconstruction process is driven by MRSI only. Otherwise, it is driven by MRI (see Equation 10).

#### 2.2.3. Mean correction

In the mean correction step of algorithm, the reconstructed values are first convolved with the PSF, which is typically a *sinc* function $\left(sinc(\pi x),where\;x=\frac{1}{\text{nominal voxel size}}=\frac{\text{no of phase encoding steps}}{\text{field of view}}\right)$ (De Graaf, 2013; Posse et al., 2013). Then, the average of the PSF corrected reconstruction values that compose the low resolution voxel *y*_*p*_ (of which *x*_*i*_ is part) must be close to the corresponding original value of the low resolution image. This corresponds to a sinc and boxcar operator for *H* in Equation (2), but it could be easily replaced by a general smoothing and downsampling operator *H*.

## 3. Experiments and results

### 3.1. Experiment 1

#### 3.1.1. Phantom data

**Figure 2:**
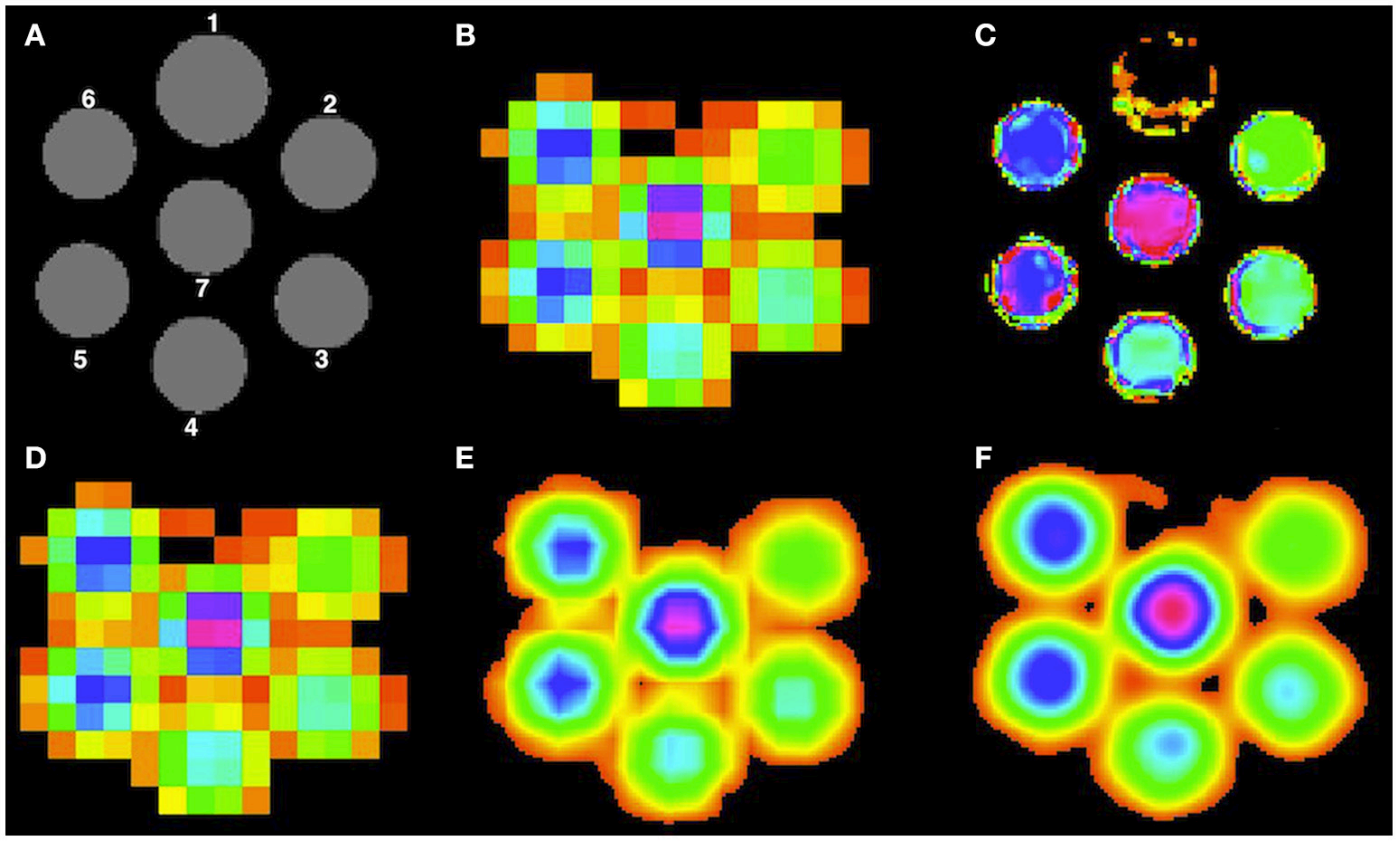
**High resolution Ace maps comparison for all methods. (A)** T1-weighted image of the phantom, **(B)** acquired low resolution Ace map. Reconstructed high resolution Ace map for **(C)** PBSR, **(D)** NN, **(E)** LIN, and **(F)** BS.

A cylindrical phantom (180 mm diameter, 168 mm height) is used, consisting of 7 small cylinders each having a diameter of 40 mm and depth of 150 mm, going through the thickness of the phantom as shown in Figure 2. The first cylinder was kept empty for orientation purposes, followed by the second cylinder having acetate (Ace) concentration of 6 mM. Then, the Ace concentration was increased by 2 mM for each next cylinder resulting in a concentration of 16 mM for the last cylinder. The rest of the empty space in the phantom was filled with water. MR imaging was performed on a 3T whole body scanner (Philips Achieva, Best, the Netherlands). The protocol contained two sequences: 3D T1-weighted fast field echo (FFE) sequence (TR = 6.6 ms, TE = 3.12 ms, FA = 9° 180 × 180 mm^2^ FOV, 3 axial slices, 0.8 × 0.8 × 10.0 mm^3^ voxel resolution) and the ^1^H MRSI point resolved spectroscopy (PRESS) sequence (TR = 1500 ms, TE = 120 ms, 300 × 300 mm^2^ FOV, 3 axial slice, 10 × 10 × 10 mm^3^ voxel resolution). MRI individual cylinder segmentation was performed manually using the Slicer tool (version 4.3.1)^3^ and the super-resolution method was then performed at three different higher resolution voxel sizes: 5 × 5 × 5 mm^3^, 2.5 × 2.5 × 2.5 mm^3^ and 1.25 × 1.25 × 1.25 mm^3^. As the phantom is homogeneous, we present in this paper only the phantom data results at 1.25 × 1.25 × 1.25 mm^3^ voxel resolution. MRSI data was quantified using the SPID software^1^ (Van Cauter et al., 2013) to obtain metabolite images for Ace.

#### 3.1.2. Evaluation criteria

We compare our method's accuracy against three conventional interpolation techniques: nearest neighbor, linear interpolation and B-splines. The nearest neighborhood (NN) function assigns the value of the new point with the closest old neighbor value. Linear interpolation (LIN) function linearly interpolates the new point between the old points. B-splines interpolation (BS) function uses piecewise cubic polynomials of degree 3 to interpolate the new point using four old points (two on each sides of the new point).

In order to avoid the need of absolute quantification for the phantom data, we compare ratios between the estimated metabolite values obtained by each method in cylinders 5 and 2, and 7 and 3, respectively, as we know that the ground truth ratio is equal to 2.

#### 3.1.3. Accuracy results on phantom dataset

Table 1 presents the quantitative results of Ace concentration ratio between cylinders 5 and 2, and 7 and 3 respectively, both at low resolution (acquired at 10 × 10 × 10 mm^3^) and at high resolution (reconstructed at 1.25 × 1.25 × 1.25 mm^3^) for all methods. On average, PBSR and LIN seem to preserve better median metabolite ratio compared to other methods. However, the visual assessment for all methods (Figure 2) shows that contrary to conventional interpolation methods, PBSR reduced the partial volume effects considerably by incorporating tissue segmentation information. Therefore, overall PBSR provides better results.

**Table 1:** **Comparison between Ace concentration at low resolution (10 × 10 × 10 mm^**3**^) and high resolution (1.25 × 1.25 × 1.25 mm^**3**^)**.

Ace ratio (median)	$\frac{cylinder\;5}{cylinder\;2}$	$\frac{cylinder\;7}{cylinder\;3}$
Low resolution	2.33	1.80
High resolution		
PBSR	1.86	1.89
NN	2.25	1.6
LIN	1.75	2.0
BS	1.83	1.75

*Cylinders 5 and 7 have twice the Ace concentration of cylinders 2 and 3, respectively*.

### 3.2. Experiment 2

#### 3.2.1. Simulated brain data

The BrainWeb^4^ phantom dataset (Cocosco et al., 1997) represents a human brain and contains three MS brain phantom images with mild, moderate and severe lesion volume. The protocol contained two sequences: 3D T1-weighted SFLASH sequence (TR = 18 ms, TE = 10 ms, FA = 30°, 181 × 181 mm^2^ FOV, 217 sagittal slices, 1 × 1 × 1 mm^3^ voxel resolution, 1% noise level and 20% RF field inhomogeneity) and custom simulated 3D FLAIR IR sequence (TR = 8000 ms, TE = 165 ms, TI = 1800 ms, FA = 90°, 181 × 181 mm^2^ FOV, 217 sagittal slices, 1 × 1 × 1 mm^3^ voxel resolution, 1% noise level and 20% RF field inhomogeneity). MRI tissue segmentation was performed using MS**metrix** (Jain et al., 2015) and these tissue segmentations were then used to simulate a high resolution N-acetylaspartate (NAA) map (1 × 1 × 1 mm^3^ voxel resolution) in the phantom brain images. In our study, the NAA concentration was chosen to be 30 (arbitrary units) in GM, 25 in WM and 20 in lesions. White Gaussian noise N(0,2) is then added to the simulated high resolution NAA map and the resulting image was downsampled using edge preserving Chebyshev type-I filter (*n* = 31) to create a low resolution NAA map (2 × 2 × 2 mm^3^ voxel resolution). MRI tissue segmentations were also downsampled by a scale factor of 2 to match the NAA map's low resolution.

#### 3.2.2. Evaluation criteria

We compare our method's accuracy against nearest neighbor, linear interpolation and B-splines interpolation techniques. For validating the accuracy on the simulated brain dataset, the global image similarity is measured with the structural similarity index (SSIM) (Wang et al., 2004) which is more compatible with the human visual assessment and is defined as:

(11) $$\begin{array}{r} SSIM\left(x,y\right)=\frac{\left(2\mu_{x}\mu_{y}+c_{1}\right)\left(2\sigma_{x,y}+c_{2}\right)}{\left(\mu_{x}^{2}+\mu_{y}^{2}+c_{1}\right)\left(\sigma_{x}^{2}+\sigma_{y}^{2}+c_{2}\right)} \end{array}$$

where (μ_*x*_, μ_*y*_) and (σ_*x*_,σ_*y*_) are the respective means and standard deviations of image *x* and *y*. In our case, the metabolite concentrations are not zero or close to zero within brain, therefore, the stability constants *c*_1_ and *c*_2_ were set to zero.

Additionally, statistical differences in the metabolite concentrations between the lesions and in white matter surrounding lesions are tested using Welsh's *t*-test (Welch, 1947) and the magnitude of this difference i.e., the effect size is measured using Cohen's *d* (Cohen, 1988).

#### 3.2.3. Accuracy results on simulated brain dataset

**Figure 3:**
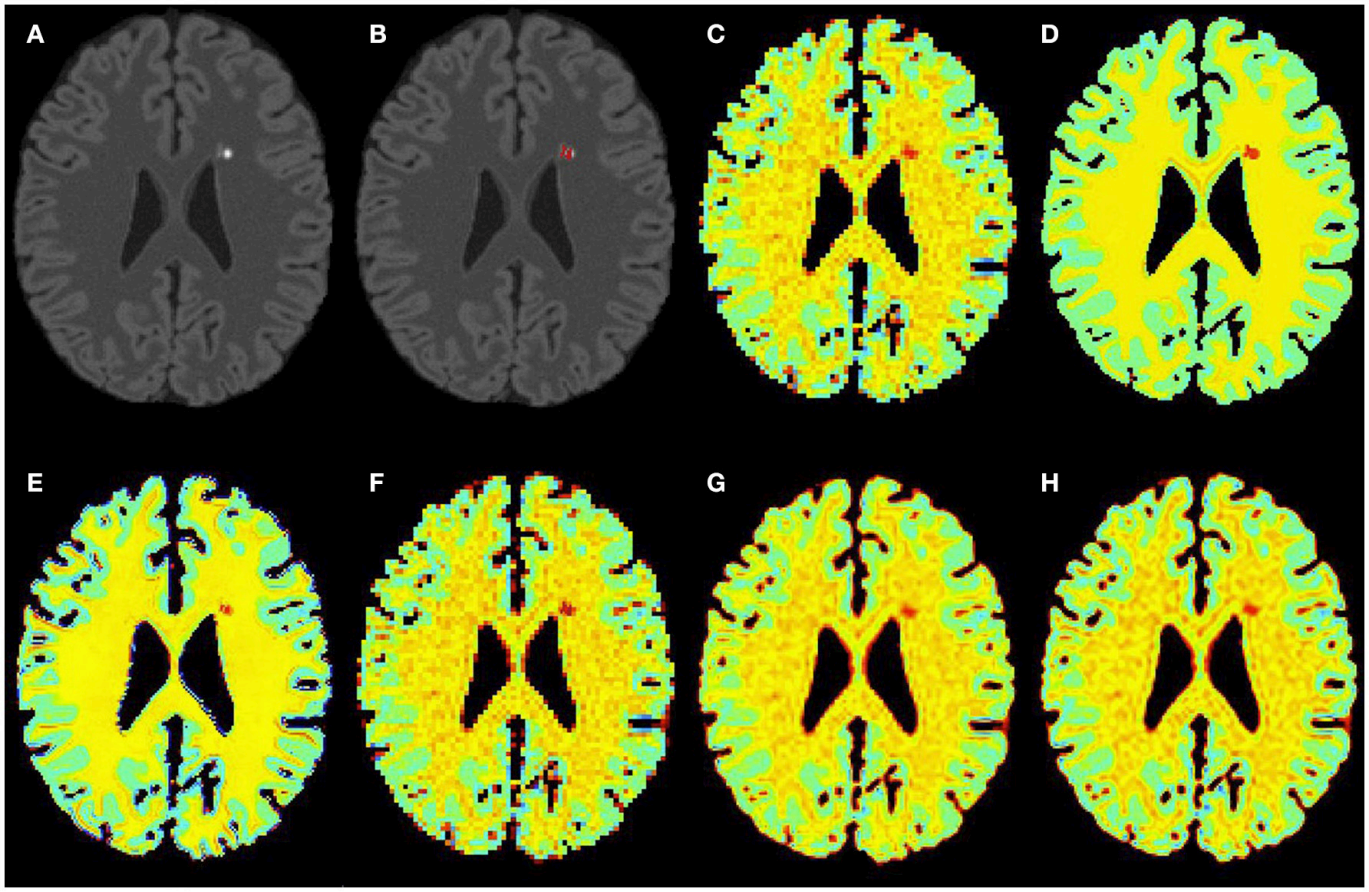
**Results on simulated mild MS subject**. **(A)** Bias corrected FLAIR image followed by **(B)** overlaid lesion segmentation, **(C)** simulated low resolution NAA map, **(D–H)** the high resolution NAA maps: **(D)** simulated, **(E)** PBSR, **(F)** NN, **(G)** LIN, and **(H)** BS. The NAA concentration increases from red to blue.

**Figure 4:**
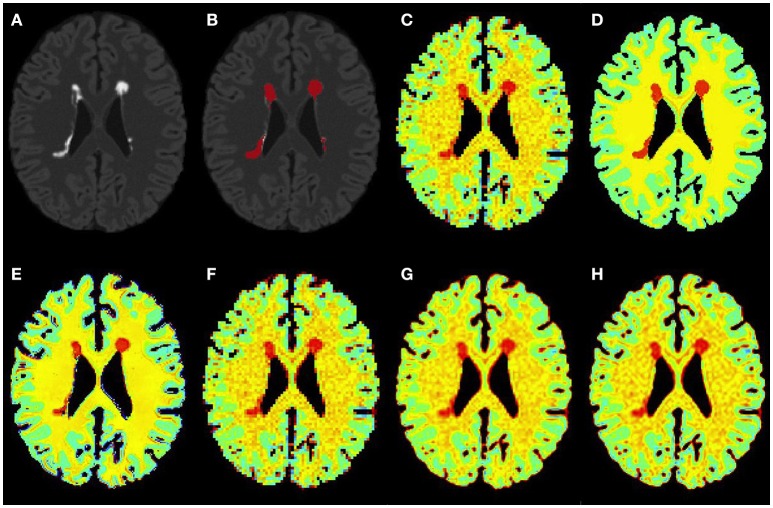
**Results on simulated moderate MS subject**. **(A)** Bias corrected FLAIR image followed by **(B)** overlaid lesion segmentation, **(C)** simulated low resolution NAA map, **(D–H)** the high resolution NAA maps: **(D)** simulated, **(E)** PBSR, **(F)** NN, **(G)** LIN, and **(H)** BS. The NAA concentration increases from red to blue.

**Figure 5:**
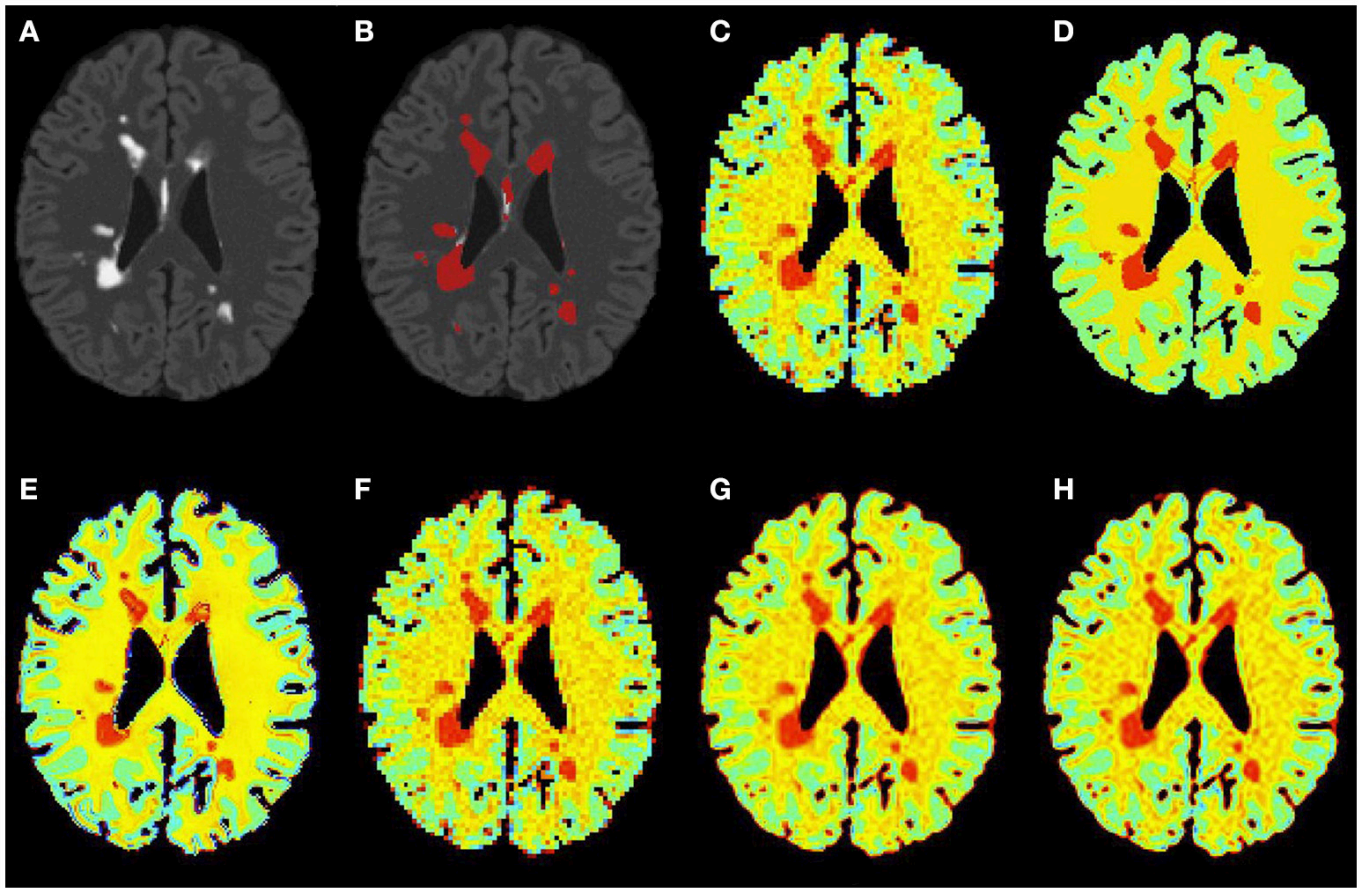
**Results on simulated severe MS subject**. **(A)** Bias corrected FLAIR image followed by **(B)** overlaid lesion segmentation, **(C)** simulated low resolution NAA map, **(D–H)** the high resolution NAA maps: **(D)** simulated, **(E)** PBSR, **(F)** NN, **(G)** LIN and **(H)** BS. The NAA concentration increases from red to blue.

Figures 3–5 show the respective visual results for all methods on mild, moderate and severe MS subjects. On close inspection, it can be seen that PBSR maintains better tissue contrast compared to other methods. For PBSR, this contrast is also more evident between lesions and their surrounding white matter, both visually and quantitatively (see Table 2). Moreover, the median value in white matter surrounding lesions for PBSR is closest to the actual value considered for WM, 25, compared to other methods. For lesions, the median value for all methods is close to 20 with slightly better effect size for PBSR. SSIM score for all methods is very high with PBSR being marginally higher.

**Table 2:** **Quantitative measures for measuring the accuracy of all methods on simulated brain datasets**.

	**NWM_*NAA*_**	**Lesions_*NAA*_**	***p*-value**	**Effect size**	**SSIM**
**MILD**					
PBSR	25.26 (24.93, 25.62)	20.34 (19.33, 22.72)	3.07 e-08	2.9	0.93
NN	24.06 (23.7, 24.11)	20.07 (18.86, 21.02)	1.65 e-06	2.12	0.93
LIN	23.75 (22.83, 24.27)	20.46 (19.94, 21.7)	2.39 e-07	2.31	0.92
BS	23.7 (22.5, 24.45)	20.07 (19.2, 21.02)	7.20 e-07	2.18	0.92
**MODERATE**					
PBSR	25.26 (23.75, 25.5)	20.38 (19.56, 22.09)	1.61 e-18	1.11	0.93
NN	23.67 (21.65, 24.99)	20.1 (19.41, 21.65)	4.71 e-19	1.08	0.92
LIN	23.11 (21.04, 24.33)	20.37 (19.6, 21.66)	2.48 e-12	0.83	0.92
BS	23.37 (20.94, 24.5)	20.25 (19.53, 21.7)	8.43 e-14	0.89	0.92
**SEVERE**					
PBSR	25.13 (22.12, 25.49)	20.47 (19.7, 21.66)	2.5 e-41	1.21	0.93
NN	22.98 (20.34, 24.83)	20.18 (19.75, 21.39)	9.54 e-36	1.11	0.92
LIN	22.94 (20.55, 24.35)	20.48 (19.77, 21.4)	1.2 e-32	1.04	0.92
BS	22.86 (20.56, 24.54)	20.28 (19.6, 21.11)	1.38 e-34	1.08	0.92

*NWM_NAA_ = NAA concentration in white matter surrounding lesions, Lesions_NAA_ = NAA concentration in lesions, p-value = Welsh's t-test p-value for testing the statistical difference between NWM_NAA_ and Lesions_NAA_, effect size = the magnitude of statistical difference, SSIM = structural similarity index. NWM_NAA_ and Lesions_NAA_ are reported as median (first quartile, third quartile)*.

### 3.3. Experiment 3

#### 3.3.1. Human dataset acquired *In vivo*

Five MS patients (4 females, 1 male, age 32–46) participated in the study at the High Field MR Centre, Department of Biomedical Imaging and Image-guided Therapy, Medical University of Vienna, Austria. This study was carried out in accordance with the recommendations of the “International Conference on Harmonization of Good Clinical Practice (ICH-GCP),” and the applicable Austrian legislation. The study was approved by the Institutional Review Board ethical committee. All subjects gave written informed consent in accordance with the Declaration of Helsinki. All measurements were performed on a 7T whole body MR scanner (Magnetom, Siemens Healthcare, Erlangen, Germany) with a 32-channel receive coil array head coil (Nova Medical, Wilmington, MA, USA). The protocol contained four sequences: (1) 3D T1-weighted MPRAGE sequence (TR = 3800 ms, TE = 3.54 ms, FA = 9°, 230 × 230 mm^2^ FOV, 208 axial slices, 0.7 × 0.7 × 0.7 mm^3^ voxel resolution), (2) 3D SPACE FLAIR sequence (TR = 8000 ms, TE = 272 ms, FA = 160°, 215 × 215 mm^2^ FOV, 160 axial slices, 0.8 × 0.8 × 0.8 mm^3^ voxel resolution), (3) low resolution ^1^H CAIPRINHA-accelerated phase-encoded FID-MRSI sequence (Strasser et al., 2016) (TR = 600 ms, TE = 1.3 ms, 220 × 220 mm^2^ FOV, single axial slice, 3.4 × 3.4 × 8.0 mm^3^ voxel resolution, an acceleration factor of 6, 5 min measurement time), and (4) high resolution ^1^H CAIPRINHA-accelerated phase-encoded FID-MRSI sequence (TR = 200 ms, TE = 1.3 ms, 220 × 220 mm^2^ FOV, single axial slice, 2.2 × 2.2 × 8.0 mm^3^ voxel resolution, an acceleration factor of 4, 6 min measurement time). Both low and high resolution MRSI data was acquired with WET (water suppression enhanced through T_1_ effects) water suppression technique. MRI tissue segmentation was performed using MS**metrix** (Jain et al., 2015) and MRSI data was quantified using the LCModel (version 6.3-1)^2^ to obtain metabolite images for NAA and myo-inositol (myo-Ins).

#### 3.3.2. Evaluation criteria

We compare our method's accuracy against nearest neighbor, linear interpolation and B-splines interpolation techniques. For validating the accuracy on the human dataset acquired *in vivo*, SSIM score is used. Also, statistical differences in the metabolite concentrations between the lesions and their neighboring WM tissue are tested using Welsh's *t*-test and the effect size is measured using Cohen's *d*.

#### 3.3.3. Accuracy on human dataset acquired *In vivo*

**Figure 6:**
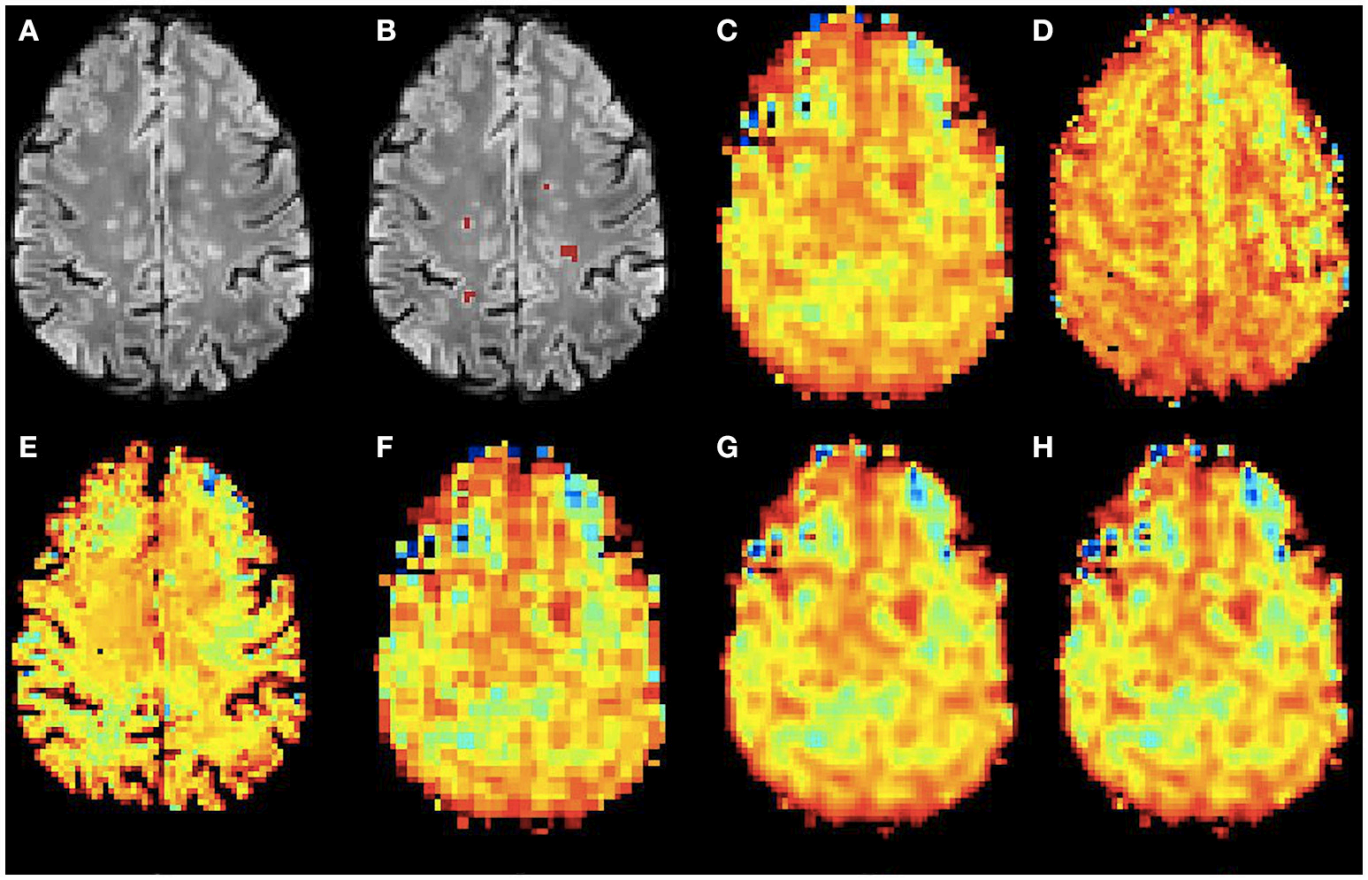
**Qualitative accuracy performance of all methods on human dataset acquired ***in vivo*** for NAA metabolite**. **(A)** Bias corrected FLAIR image followed by **(B)** overlaid lesion segmentation, **(C)** the acquired low resolution NAA map; **(D–H)** the high resolution NAA maps: **(D)** acquired, **(E)** PBSR, **(F)** NN, **(G)** LIN and **(H)** BS. The NAA concentration increases from red to blue.

**Figure 7:**
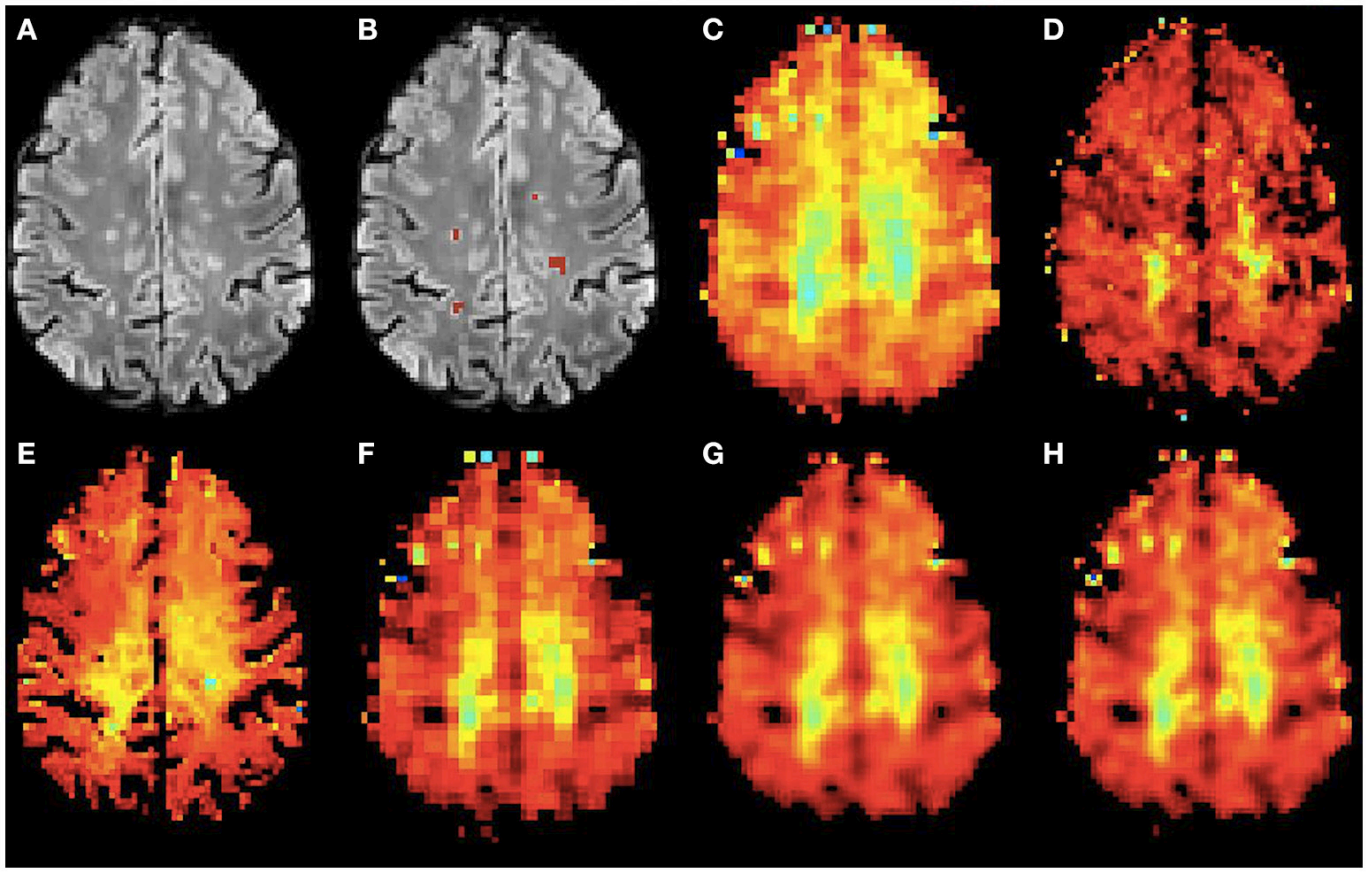
**Qualitative accuracy performance of all methods on human dataset acquired ***in vivo*** for myo-Ins metabolite**. **(A)** Bias corrected FLAIR image followed by **(B)** overlaid lesion segmentation, **(C)** the acquired low resolution myo-Ins map; **(D–H)** the high resolution myo-Ins maps: **(D)** acquired, **(E)** PBSR, **(F)** NN, **(G)** LIN and **(H)** BS. The myo-Ins concentration increases from red to blue.

Figures 6, 7 show the high resolution NAA and myo-Ins maps respectively, for all methods on a representative example. In contrast to the results on the simulated brain dataset, visually it is very difficult to see a correlation between any method and the acquired high resolution maps. However, quantitatively, a contrast between lesions and surrounding white matter can be computed (Table 3). The acquired high resolution shows a significant contrast between lesions and their surrounding WM, which is not seen at acquired low resolution. For NAA, this significant contrast can only be seen in PBSR. For myo-Ins, this significant contrast is highest for LIN followed by BS, NN and PBSR. The average SSIM score is highest for PBSR for both NAA and myo-Ins compared to the other methods, which have equal average SSIM scores.

**Table 3:** **Results on human datasets acquired ***in vivo*****.

	**NWM_*NAA*_**	**Lesions_*NAA*_**	***p*-value**	**Effect size**	**aSSIM**
**NAA**					
ALR	15.18 (14.47, 16.87)	15.62 (13.39, 18.02)	0.8	−0.07	NA
AHR	13.41 (12.48, 15.0)	12.99 (10.99, 14.85)	0.03	0.31	NA
PBSR	16.85 (15.99, 17.6)	15.6 (13.82, 17.27)	2.0 e-05	0.64	0.18
NN	16.23 (14.82, 17.34)	15.75 (13.62, 17.61)	0.35	0.14	0.13
LIN	16.1 (14.93, 17.26)	15.71 (13.85, 17.35)	0.38	0.13	0.13
BS	16.15 (14.79, 17.33)	15.49 (13.64, 17.5)	0.3	0.15	0.13
**myo-INS**					
ALR	10.8 (10.09, 12.69)	11.88 (10.9, 14.48)	5.0 e-02	−0.57	NA
AHR	6.03 (4.78, 7.86)	11.48 (9.13, 13.15)	4.3 e-16	−1.63	NA
PBSR	8.07 (6.78, 9.39)	10.59 (9.89, 11.79)	3.0 e-08	−1.04	0.2
NN	9.17 (7.77, 9.98)	11.97 (10.5, 13.35)	1.9 e-09	−1.15	0.18
LIN	9.11 (7.89, 10.11)	11.98 (10.82, 13.25)	4.7 e-11	−1.27	0.18
BS	9.12 (7.86, 10.15)	11.88 (10.72, 13.35)	1.2 e-10	−1.23	0.18

*NWM_NAA_ = NAA concentration in white matter surrounding lesions, Lesions_NAA_ = NAA concentration in lesions, p-value = Welsh's t-test p-value for testing the statistical difference between NWM_NAA_ and Lesions_NAA_, effect size = the magnitude of statistical difference, aSSIM = averaged structural similarity index over 5 subjects. NWM_NAA_ and Lesions_NAA_ are reported as median (first quartile, third quartile). ALR, acquired low resolution map; AHR, acquired high resolution map*.

## 4. Discussion and conclusion

In this paper, we presented a patch-based super-resolution method for upsampling the low resolution quantified metabolite maps. The method incorporates the high resolution spatial information from T1-weighted and FLAIR MR images in guiding the reconstruction process. The method iteratively estimates and corrects for the metabolite concentration at a high resolution. In contrast to the k-space based techniques where anatomically derived information is used to improve the spectral quality of high resolution MRSI data, in our case, the method reconstructs each central voxel *x*_*i*_ using a weighted average of voxels that have similar tissue composition as the central voxel in the search volume. Moreover, as MRI and MRSI have complementary information, separate weights are defined for each modality which are regulated with a parameter α(*x*_*i*_). Depending on the application, α(*x*_*i*_) gives the flexibility to control the influence of each modality in the reconstruction process. In this paper, when α(*x*_*i*_) = 1, the reconstruction is guided by MRSI only resulting in a smooth image and when α(*x*_*i*_) = 0, the reconstruction is guided by MRI alone resulting in a better tissue contrast compared to conventional interpolation techniques (see Tables 2, 3). The reconstruction quality is affected by the potential registration errors and the reliability of the input quantified metabolite map. Additionally, the method could not be expected to recover small scale features that were not sufficiently picked up by the low resolution measurement. On a similar note, the use of tissue segmentations to guide the reconstruction might affect metabolite values at tissue interfaces. In particular, through such an effect, some hyper intense voxels are introduced near the brain boundary, requiring further investigation. Finally, if the difference between the image resolution of MRI and MRSI images is big (scale factor more than 4), then the downsampled tissue segmentations for super-resolution process have high partial volume effect and thus do not carry much information on tissue type. That is why in this study, we limited the in-plane resolution of acquired low resolution MRSI to ~3.4 × 3.4 mm^2^. Our method can also be implemented in a multi-scale fashion. However, validating such high resolution reconstructed image would be a problem in case of real datasets (e.g., Experiment 3) where acquiring such high resolution image is not possible due to practical limitations. Indeed, multi-scale super-resolution method can easily be tested on a simulated data, and, as our results show better tissue contrast at the current high resolution, we expect similar or better results at even higher resolution. Our main aim in this paper was to validate the method on real datasets and thus we opted not to use the multi-scale super-resolution.

Among the methods proposed in the literature for super-resolution, our approach has some similarities to Rousseau and The Alzheimer's Disease Neuroimaging Initiative (2010), which is also based on patch-based framework. In contrast with Rousseau and The Alzheimer's Disease Neuroimaging Initiative (2010), which uses a neighborhood averaging strategy to define MRI weights, we used brain tissue segmentations. In Rousseau and The Alzheimer's Disease Neuroimaging Initiative (2010), α(*x*_*i*_) defines the correlation between high resolution and low resolution images and is adapted in each iteration. In our case, α(*x*_*i*_) is defined using binarized lesion segmentation and is fixed throughout the process.

One of the biggest problems that we encountered in this study was having ideal data for method validation. Challenges include (a) absence of high resolution ground truth data, (b) lack of a publicly available datasets, and (c) difference between low and high resolution acquired MRSI data. Acquiring high resolution ground truth data in reality is not feasible because low metabolite concentration requires large voxel size to capture sufficient signal, and prolonged acquisition time. Although several group studies have reported metabolite concentration in normal and pathological cases like MS (Sajja et al., 2009; Bogner et al., 2012), conclusions are not easily generalizable when it comes to individual patients. Lack of a standardized acquisition protocol for high spatial resolution MRSI hampers the creation of publicly available datasets against which state-of-the-art methods' performances can be compared. Moreover, most of the state-of-the-art super-resolution methods for MRSI improve MRSI data using sophisticated reconstruction techniques (k-space based), which make their reproducibility for comparison very difficult. However, from methodological point of view, our method requires fewer variables to be optimized simultaneously which simplify the underlying optimization problem. For example, in k-space based methods, the MRI spatial parameters (e.g., bias field inhomogeneity, tissue class information) and the MRSI spectral parameters (e.g.,metabolite amplitudes, B_0_ shifts, zero-order phase and lineshape parameters) have to be optimized simultaneously, which may result in a sub-optimal solution. In our case, we deal with the MRI spatial parameters and MRSI spectral parameters separately. Bias field correction and tissue class segmentation are performed by MSmetrix (2.1.1) and the interaction between neighborhood voxels is exploited in the proposed super-resolution method. The spectral parameters are estimated by state-of-the-art metabolite quantification methods such as LCmodel or SPID.

In human MRSI data acquired *in vivo*, the median metabolite concentrations acquired at low resolution are greater than those at higher resolution (see Table 3), although the same quantification method [LCModel]^2^ and parameters have been used to quantify metabolic maps at both resolutions. This generates a degree of uncertainty in the interpretation of the performance analysis. This issue was not present in the simulated data, which explains a significant decrease in the SSIM score from simulated brain dataset's results (see Table 2) to human dataset acquired *in vivo* (see Table 3). This decrease can also be explained by the fact that simulated low resolution MRSI data has twice the resolution of low resolution MRSI human dataset acquired *in vivo*, resulting in a loss of structural information. Finally, it may seem like the conventional interpolation methods provide better results than PBSR for myo-Ins (see Table 3), however, this is not completely true. Four out of five MS subjects in the human dataset acquired *in vivo*, have low lesion load in the acquired MRSI plane and the remaining subject has high lesion load. If this subject is removed, none of the methods show any statistical difference in the metabolite concentrations between the lesions and their neighboring WM tissue for myo-Ins. However, this statistical difference still holds true for NAA. Tuning the parameter α(*x*_*i*_) may address this issue and will be explored.

In conclusion, we presented a patch-based super-resolution approach for upsampling the low resolution quantified metabolites maps using T1-weighted and FLAIR MR images. The proposed method preserves tissue contrast and structural information compared to conventional interpolation methods, and matches well with the trend of acquired high resolution MRSI. These results suggest that the method has potential for clinically relevant neuroimaging applications.

## Author contributions

SJ, DMS, SV, FM, DS contributed to the design and analysis of the work; FS, GH, WB, SW contributed to the data acquisition; SJ and DMS wrote the paper; all authors revised the manuscript critically for important intellectual content.

## Funding

This research received funding from the TRANSACT (EU-FP7-PEOPLE-2012-ITN-316679); CENTER-TBI (FP7-COOPERATION-2013-602150); BRAINPATH (FP7-PEOPLE-2013-IAPP-612360); FFG Bridge Early Stage Grant #846505; EPSRC (UK) Grant ref:EP/M005909/1, and the research support from the NIHR/WT Clinical Research Facility, at the University of Manchester and the Manchester Academic Health Sciences Centre.

### Conflict of interest statement

The authors declare that the research was conducted in the absence of any commercial or financial relationships that could be construed as a potential conflict of interest.

## Footnotes

^1^SPID (Online; accessed 20 Oct. 2016). http://homes.esat.kuleuven.be/~biomed/software.php

^2^LCModel (Online; accessed 20 Oct. 2016). http://s-provencher.com/lcmodel.shtml

^3^3D Slicer (Online; accessed 20 Oct. 2016). www.slicer.org.

^4^BrainWeb (Online; accessed 20 Oct. 2016). http://www.bic.mni.mcgill.ca/brainweb.
